# Differences in the prognosis of HPV16-positive patients with squamous cell carcinoma of head and neck according to viral load and expression of P16

**DOI:** 10.1007/s00432-017-2531-2

**Published:** 2017-10-17

**Authors:** Beata Biesaga, Anna Mucha-Małecka, Anna Janecka-Widła, Marta Kołodziej-Rzepa, Sława Szostek, Dorota Słonina, Aleksandra Kowalczyk, Krzysztof Halaszka, Marcin Przewoźnik

**Affiliations:** 1 Department of Applied Radiobiology, Maria Sklodowska-Curie Institute-Oncology Center, Cracow Branch, 11 Garncarska Street, 31-115 Cracow, Poland; 2 Department of Radiation Oncology, Maria Sklodowska-Curie Institute-Oncology Center, Cracow Branch, 11 Garncarska Street, 31-115 Cracow, Poland; 3 Department of Surgical Oncology, Maria Sklodowska-Curie Institute-Oncology Center, Cracow Branch, 11 Garncarska Street, 31-115 Cracow, Poland; 4 Department of Virology, Chair of Microbiology, Jagiellonian University Medical College, 18 Czysta Street, 31-121 Cracow, Poland; 5 Department of Tumour Pathology, Maria Sklodowska-Curie Institute-Oncology Center, Cracow Branch, 11 Garncarska Street, 31-115 Cracow, Poland

**Keywords:** HNSCCs, HPV16, Viral load, P16 expression, Prognosis

## Abstract

**Purpose:**

To evaluate the impact of HPV16 load (VL—the number of virus genome copies per cell) and P16 expression on prognosis of patients with squamous cell carcinomas (SCCs) of head and neck (HN).

**Materials and methods:**

HPV16 presence was assessed in the group of 109 patients with HNSCCs by quantitative polymerase chain reaction (qPCR). VL (assessed by qPCR) and P16 expression (evaluated by immunohistochemistry) were analysed only in the subgroup of HPV16-positive tumours. These features were correlated with 5-year overall survival (OS) and disease-free survival (DFS).

**Results:**

HPV16 infection was found in 36 tumours (33.0%). Virus-positive patients had better OS and DFS than those without infection (*P* = 0.041 and 0.005). Among HPV16-positive HNSCCs, 18 (50.0%) had higher VL (median value > 6764.3 copies/cell) and 25 (73.5%) P16 over expression. The significant differences in OS and DFS (*P* = 0.008 and 0.004) were noticed according to VL, wherein 100% DFS was found for patients with higher VL. According to P16 expression, significant difference was found only for OS (*P* = 0.020). In multivariate analysis, VL (*P* = 0.045; HR = 2.795; CI 0.121–1.060) and the level of smoking (*P* = 0.023, HR = 2.253; CI 1.124–4.514) were independent factors affecting DFS of HPV16-positive patients.

**Conclusion:**

On the basis of viral load, it is possible to differentiate prognosis of patients with HPV16-positive HNSCCs. In this subgroup, viral load has stronger prognostic potential than P16 expression.

## Introduction

Epidemiological studies have shown important role of high-risk (HR) human papillomavirus (HPV) in development of some types of squamous cell carcinomas (SCCs) of head and neck (HN) (Castellsagué et al. 2017). This infection (mostly HPV16) is predominantly observed in cancers of the oral cavity, oropharynx and larynx (Castellsagué et al. 2017). The meta-analyses, covering 99 studies, have found more favourable prognosis for patients with HPV-positive HNSCCs than for those without infection (Ragin and Taioli 2007; Dayyani et al. 2010; O’Rorke et al. 2012; Liu et al. 2016). However, the values of the hazard ratios from these analyses 0.30 (O’Rorke et al. 2012)–0.62 (Ragin and Taioli 2007) show that in more than 40% of HPV-infected patients progression of cancer disease is observed. Their identification is particularly important nowadays, because of ongoing trials concerning de-escalation of anticancer treatment in patients with HPV-positive HNSCCs (Mirghani et al. 2015).

Researchers attempt to identify patients with HPV-positive HNSCCs and worse prognosis on the basis of viral load (VL—the number of HPV16 copies expressed per sample or per single cell). However, results of these studies are ambiguous, as in some of them positive prognosis is associated with higher VL (Mellin et al. 2002; Cohen et al. 2008; Worden et al. 2008; Holzinger et al. 2012), while in others, opposite correlation is found (Huang et al. 2014). On the other hand, some authors have shown significant positive correlation between VL and P16 overexpression (Rödel et al. 2015) and between higher VL and expression of HPV E6/E7 mRNA (indicating transcriptionally active HPV infection) (Jung et al. 2010; Holzinger et al. 2012; Deng et al. 2013). Our suggestion is that high VL reflects active infection. Hence, the present study aims to: (1) assess HPV16 presence by amplification of virus *E6* gene fragment in quantitative polymerase chain reaction (qPCR) in 109 patients with HNSCCs from Cracow area and (2) analyse the influence of VL (HPV16 genome copies calculated per single cell) and P16 expression on 5-year overall survival (OS) and 5-year disease-free survival (DFS) in the subgroup of HPV-positive patients.

## Materials and methods

### Patients

The study was performed in 109 patients with SCCs of oral cavity, oropharynx, hypopharynx and larynx, with no distant metastasis at the moment of diagnosis and treated, between 2007 and 2014, in Maria Sklodowska-Curie Memorial Cancer Center and Institute of Oncology, Cracow Branch. The study was approved by the Ethical Committee at the Regional Medical Chamber in Cracow (Poland) on 19 September 2012 (109/KBL/OIL/2012) on 19 September 2012 (109/KBL/OIL/2012). No informed consents from patients were required, because during the study no direct contact with patients and use of personal data were necessary. All samples were anonymized.

### Histopathological verification of formalin-fixed and paraffin-embedded cancer specimens, DNA isolation

For 109 patients, formalin-fixed and paraffin-embedded (FFPE) cancer specimens (obtained during surgery or biopsy) were collected. They have been subjected to histopathological reverification, which included: tumour histology (squamous cell carcinoma), histologic grade and degree of keratinization. Selected for further analysis were these FFPE, in which tumour component covered > 50% of the slide area.

From FFPE selected by pathologists DNA was isolated using ReliaPrep™ FFPE gDNA Miniprep System (Promega, Madison, USA) based on manufacturer’s suggestions with our own modification. The procedure was detailed previously (Biesaga et al. 2016).

### HPV16 presence and its load

The HPV16 presence was determined on the basis of amplification of 81-bp fragment of virus *E6* gene with primers (F: GAG AAC TGC AAT GTT TCA GGA CC, R: TGT ATA GTT GTT TGC AGC TCT GTG C) and TaqMan probe (6FAM-CAG GAG CGA CCC AGA AAG TTA CCA CAG TT-TAMRA), synthesized by Thermo Fisher Scientific, Waltham, USA, as previously described in detail (Biesaga et al. 2012). In brief, amplification was carried out in a 25 µl mixture containing: 12.5 µl of Fast Universal PCR Master Mix (2 X), 100 nM of each primer, 300 nM of probe and 80 ng of DNA template. Thermal cycling (ViiA 7, Thermo Fisher Scientific, Waltham, USA) consisted of initial denaturation (20 s, 95°C) and 45 cycles of 3 s at 95 °C and 30 s at 60 °C. Each sample was tested in duplicate.

To each sample set analysed for HPV16 detection, a series of tenfold dilutions of HPV16 plasmid (ATCC, USA), containing from 5 × 10^8^ to 5 × 10^12^ HPV16 copies, was added. This allowed to draw a standard curve (Ct vs the number of virus copies), on which Ct values obtained for clinical samples were plotted and the number of HPV16 genome copies was calculated. Additionally, to evaluate VL, a number of cells in each sample were analysed. For this purpose, each DNA was subjected to qPCR for amplification of 139-bp fragment of *β*-*actin* gene using TaqMan^®^ Gene Expression Assay (Thermo Fisher Scientific, Waltham, USA), with mix of specific primers and MGB probe. Amplification was carried out in reaction volume of 20 µl, containing: 10 µl of Fast Universal PCR Master Mix (2 X) (Thermo Fisher Scientific, Waltham, USA), 1 µl of TaqMan Gene Expression Assay and 50 ng of DNA template. qPCR was initiated with 20-s incubation at 95 °C; then, 40 cycles at 95 °C for 1 s and 60 °C for 20 s were applied (ViiA 7, Thermo Fisher Scientific, Waltham, USA). Two replicates were used per sample. To generate standard curve and calculate the number of cells in sample, for each assay, serial tenfold dilutions of human genomic DNA (Roche Diagnostics, GmbH, Germany), containing from 1 to 114,200 *β*-*actin* copies, were added. HPV16 VL was calculated as the number of virus copies per cell, assuming that two copies of *β*-*actin* gene correspond to one cell (Mellin et al. 2002).

### P16 immunostaining

**Fig. 1:**
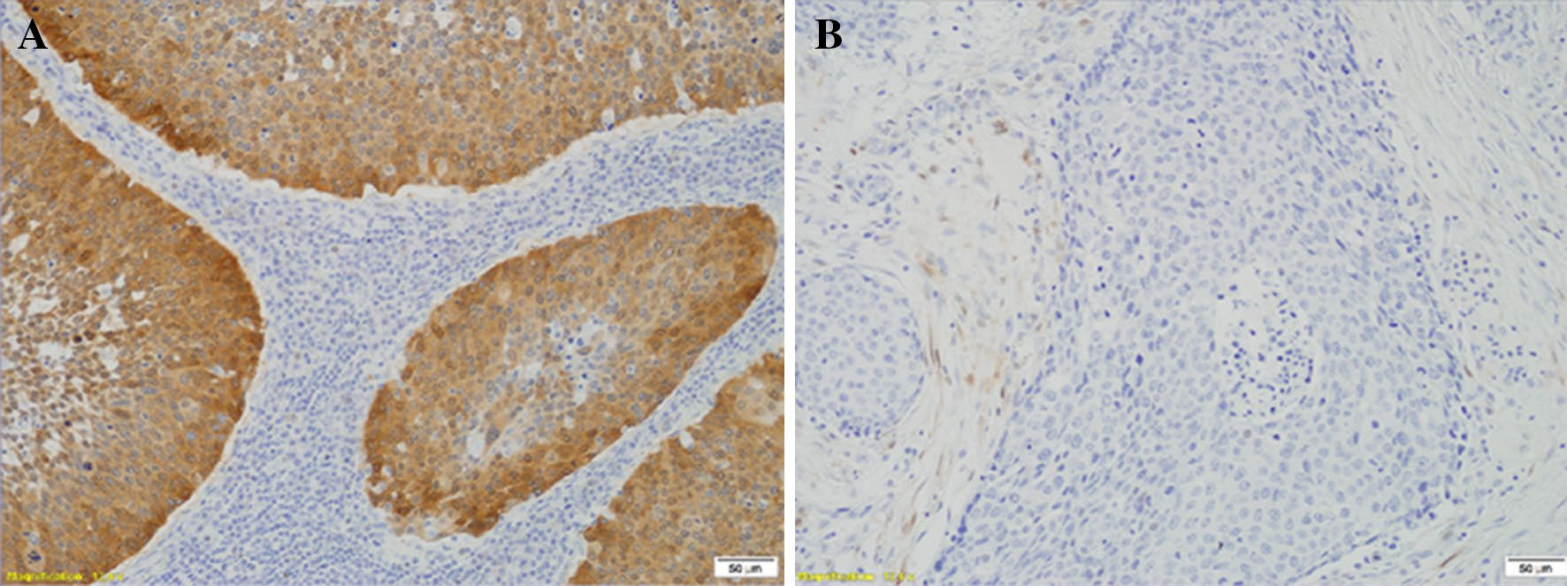
Immunohistochemical staining of P16 using CINtec^®^ Histology Kit (Roche, Heidelberg, Germany) in formalin-fixed paraffin-embedded samples of head and neck cancers. **a** Tumour with P16 overexpression defined by Lewis et al. (2012) as follows: > 75% of positive staining cells or > 50% staining with > 25% confluent positive staining areas. **b** Tumour with lack of P16 overexpression

P16 immunostaining was performed in the subgroup of HPV16-positive tumours using CINtec^®^ Histology Kit (Roche, Heidelberg, Germany) according to the manufacturer’s procedure. Briefly, after deparaffinization and rehydration, antigen retrieval (96 °C for 10 min) and quenching of endogenous peroxidases procedures were applied. Incubations with primary antibody and visualization system were carried out for 30 min. The reaction was visualized with DAB as a chromogen. Slides were counterstained using Mayer’s haematoxylin. To each set of staining-negative (absence of primary antibody) and staining-positive (cervical cancer with known P16 strong reaction) controls were added. Immunopositivity was defined according to Lewis et al. (2012) as follows: > 75% of positive staining cells or > 50% staining with > 25% confluent areas of positive staining (Fig. 1).

### Statistical analysis

Descriptive statistics were used to determine mean and median of continuous variables and standard errors of means (SE). Student’s *t* test was applied to establish the significance of differences between means. Associations between categorical variables were analysed using Pearson Chi-square test. To analyse prognostic potential, two endpoints were adopted: OS (time from the end of therapy until death from any cause within 5 years after completing the treatment) and DFS (time from the end of therapy until the first documented evidence of recurrent disease—treatment failure, locoregional recurrence, distant metastasis, within 5 years after completing the treatment). We decided to define OS and DFS from the date of treatment completing and not from the date of initial diagnosis in order to eliminate differences between individual patients in the period of time between diagnosis and the start of treatment and in the total treatment time. Survival curves were obtained using Kaplan–Meier estimates, and differences between groups were tested by the log-rank test. Univariate and multivariate survival analyses were carried out according to the Cox proportional hazards model. Multivariate analysis included all the parameters for which in univariate analysis, statistically significant influence on survival was observed. All statistical tests were two-sided, and *P* < 0.050 was considered significant. Statistical analyses were carried out using Statistica version 10.0 program.

## Results

### Patients

The mean and median age of 109 patients were 57.3 ± 0.9 and 58 years. There were 9 (8.3%) patients with tumours in stage II, 22 (20.2%) in III and 78 (71.6%) in IV. The detailed patients and tumour characteristics are summarized in Table 1.

**Table 1:** Clinical and histopathological features according to HPV16 presence assessed by amplification of virus *E6* gene fragment in quantitative polymerase chain reaction in the group of 109 patients with squamous cell carcinomas of head and neck

Feature	All *N* (%)^a^	HPV16^+^ *N* (%)	HPV16^−^ *N* (%)	*P* value (test *χ* ^2^)
All	109 (100.0)	36 (33.0)	73 (67.0)	
Age				
≤ 58 years^b^	51 (46.8)	12 (33.3)	39 (53.4)	0.048
> 58 years	58 (53.2)	24 (66.7)	34 (46.6)	
Gender				
Female	22 (20.2)	9 (25.0)	13 (17.8)	0.379
Male	87 (79.8)	27 (75.0)	60 (82.2)	
Status in the Karnofsky scale				
≤ 80%	49 (45.0)	15 (41.7)	34 (46.6)	0.628
> 80%	60 (55.0)	21 (58.3)	39 (53.4)	
Localization				
Oral cavity	24 (22.0)	6 (16.6)	18 (24.7)	0.501
Oropharynx	68 (62.4)	26 (72.2)	42 (57.5)	
Hypopharynx	7 (6.4)	2 (5.6)	5 (6.8)	
Larynx	10 (9.2)	2 (5.6)	8 (11.0)	
*T* stage				
1	2 (1.8)	0 (0.0)	2 (2.7)	0.211
2	24 (22.0)	9 (25.0)	15 (20.6)	
3	60 (55.1)	23 (63.9)	37 (50.7)	
4	23 (21.1)	4 (11.1)	19 (26.0)	
*N* stage				
0	19 (17.4)	5 (13.9)	14 (19.1)	0.820
1	18 (16.5)	5 (13.9)	13 (17.8)	
2	61 (56.0)	22 (61.1)	39 (53.4)	
3	11 (10.1)	4 (11.1)	7 (9.6)	
Grade				
1	40 (36.7)	12 (33.3)	28 (38.4)	0.641
2	57 (52.3)	21 (58.3)	36 (49.3)	
3	12 (11.0)	3 (8.3)	9 (12.3)	
Keratinization				
Yes	62 (56.9)	15 (41.7)	47 (64.0)	0.024
No	47 (43.1)	21 (58.3)	26 (36.0)	
The level of smoking—Brinkman index^c^				
≤ 520^b^	54 (49.5)	20 (55.6)	34 (46.6)	0.378
> 520	55 (50.5)	16 (67.6)	39 (53.4)	
The level of drinking^d^				
Low	50 (45.9)	20 (55.6)	30 (41.1)	0.154
High	59 (54.1)	16 (44.4)	43 (58.9)	
Treatment				
Definitive CRT or surgery + CRT	46 (42.2)	19 (52.8)	27 (37.0)	0.289
Definitive RT or surgery + RT	38 (34.9)	10 (27.8)	28 (38.3)	
Induction CT + definitive RT	25 (22.9)	7 (19.4)	18 (24.7)	
Treatment outcome				
Regression of cancer disease	71 (65.1)	28 (77.8)	43 (58.9)	0.151
Treatment failure	6 (5.5)	1 (2.8)	5 (6.9)	
Local recurrence	22 (20.2)	6 (16.7)	16 (21.9)	
Distant metastases	10 (9.2)	1 (2.8)	9 (12.3)	
Survival				
Alive at the last follow-up	74 (67.9)	28 (77.8)	46 (63.0)	0.291
Death from cancer disease	19 (17.4)	4 (11.1)	15 (20.6)	
Death from others reasons	16 (14.7)	4 (11.1)	12 (16.4)	

^a^Row percentage

^b^Median value

^c^Number of cigarettes per day × years of smoking

^d^Low level of drinking—no alcohol and occasional drinkers (at most two drinks a day, especially with a meal) high level of drinking—more than 15 drinks of high percentage alcohol in a week and alcoholics

Most patients (46) were subjected to concurrent chemoradiotherapy (CRT) as a definitive or post-operative treatment. The total dose of radiotherapy (RT) ranged from 28 to 70 Gy (mean 66.1 Gy ± 1.5), applied in 14–35 fractions of 2.0–2.2 Gy. During RT, cisplatin (CisPt) was administrated according to two schemes: (1) 100 mg CisPt/m^2^ every 3rd week of RT in 2–3 courses or (2) 40 mg CisPt/m^2^ every week of RT in 3–6 courses (depending on patient’s condition and the severity of early normal tissue reactions). For 38 patients, RT (alone or post-operative) was applied (total dose of 20.0–74.0 Gy, fraction dose: 1.8–4.0 Gy, number of fractions: 5–40). In turn, 25 patients were treated with induction chemotherapy (CisPt + 5-fluorouracil + taxanes), followed by RT (total dose: 20–70 Gy, fraction dose: 2–4 Gy, number of doses: 5–35).

The mean follow-up time was 37.8 months (1–114 months). Among 109 patients, 74 were alive at the time of the study, 19 died from cancer disease and 16 from others reasons, mainly cardiovascular disease. Regression of cancer was noticed in 71 persons (65.1%) and progression (treatment failure, locoregional recurrence, distant metastases) occurred in 38 patients (34.9%), from 0 to 88 months after completing treatment (mean and median: 16.3 months ± 3.2 and 10 months). Because of low number of distant metastasis (*n* = 10), we did not decide to assess metastasis-free survival.

### HPV16 infection, viral load, P16 expression—correlation with clinical and histopathological data

Among 109 tumours, HPV16 infection (assessed on the basis of *E6* gene fragment amplification) was found in 36 cases (33.0%) (Table 1). Infection was significantly more common in older patients, as well as in oropharynx and in non-keratinizing tumours. The distribution of HPV16-positive and HPV16-negative tumours was not significantly related to other clinical features studied.

In the subgroup of 36 HPV16-positive cancers, the mean and median values of VL were 90 407.8 copies/cell ± 62 493.7 (SE) and 6764.3 copies/cell (range 0.9–2244, 936.0). HPV16-positive tumours were grouped, according to VL median value, as those with lower (≤ 6764.3 copies/cell, *n* = 18) and with higher VL (> 6764.3 copies/cell, *n* = 18) (Table 2). Most patients having tumours with higher VL did not suffer from alcohol abuse and were treated with CRT. No other significant relations were found between VL and clinical or histopathological features.

**Table 2:** Clinical and histopathological features of 36 patients with HPV16-positive HNSCC (assessed by amplification of *E6* gene fragment) according to viral load and P16 immunostaining

Feature	Viral load ≤ 6764.3 copies/cell^a^ *N* (%)^b^	Viral load > 6764.3 copies/cell *N* (%)	*P* value (test *χ* ^2^)	Lack of P16 overexpression *N* (%)	P16 overexpression *N* (%)	*P* value (test *χ* ^2^)
All	18 (50.0)	18 (50.0)		9 (26.5)	25 (73.5)	
Age						
≤ 58 years^a^	6 (33.3)	6 (33.3)	1.000	4 (44.4)	7 (28.0)	0.366
> 58 years	12 (66.7)	12 (66.7)		5 (55.6)	18 (72.0)	
Gender						
Female	4 (22.2)	5 (27.8)	0.700	1 (11.1)	17 (68.0)	0.223
Male	14 (77.2)	13 (72.2)		8 (88.9)	8 (32.0)	
Status in the Karnofsky scale						
≤ 80%	9 (50.0)	6 (33.3)	0.310	3 (33.3)	10 (40.0)	0.724
> 80%	9 (50.0)	12 (66.7)		6 (66.7)	15 (60.0)	
Localization						
Oral cavity	3 (16.7)	3 (16.7)	0.202	2 (22.2)	4 (16.0)	0.003
Oropharynx	11 (61.1)	15 (83.3)		3 (33.4)	21 (84.0)	
Hypopharynx	2 (11.1)	0 (0.0)		2 (22.2)	0 (0.0)	
Larynx	2 (11.1)	0 (0.0)		2 (22.2)	0 (0.0)	
*T* stage						
1	0 (0.0)	0 (0.0)	0.985	0 (0.0)	0 (0.0)	0.861
2	4 (22.2)	5 (27.8)		3 (33.3)	6 (24.0)	
3	12 (66.7)	11 (61.1)		5 (55.6)	16 (64.0)	
4	2 (11.1)	2 (11.1)		1 (11.1)	3 (12.0)	
*N* stage						
0	4 (22.2)	1 (5.6)	0.072	4 (44.4)	1 (4.0)	0.016
1	0 (0.0)	5 (27.8)		0 (0.0)	5 (20.0)	
2	12 (66.7)	10 (55.6)		5 (55.6)	16 (64.0)	
3	2 (11.1)	2 (11.1)		0 (0.0)	3 (12.0)	
Grade						
1	7 (38.9)	5 (27.8)	0.700	4 (44.4)	8 (32.0)	0.597
2	10 (55.6)	11 (61.1)		5 (55.6)	15 (60.0)	
3	1 (5.5)	2 (11.1)		0 (0.0)	2 (8.0)	
Keratinization						
Yes	7 (38.9)	8 (44.4)	0.735	5 (55.6)	15 (60.0)	0.816
No	11 (61.1)	10 (55.6)		4 (44.4)	10 (40.0)	
The level of smoking—Brinkman index^c^						
≤ 520^a^	8 (44.4)	12 (66.7)	0.180	4 (44.4)	16 (64.0)	0.307
> 520	10 (55.6)	6 (33.3)		5 (55.6)	9 (36.0)	
The level of drinking^d^						
Low	4 (22.2)	16 (88.9)	0.000	1 (11.1)	18 (72.0)	0.002
High	14 (77.8)	2 (11.1)		8 (88.9)	7 (28.0)	
Viral load						
≤ 6764.3 copies/cell^a^	–	–		9 (100.0)	7 (28.0)	0.000
> 6764.3 copies/cell	–	–		0 (0.0)	18 (72.0)	
Treatment						
Definitive CRT or surgery + CRT	5 (27.8)	14 (77.8)	0.009	3 (33.3)	16 (64.0)	0.283
Definitive RT or surgery + RT	7 (38.9)	3 (16.7)		4 (44.4)	6 (24.0)	
Induction CT + definitive RT	6 (33.3)	1 (5.5)		2 (22.2)	3 (12.0)	
Treatment outcome						
Regression of cancer disease	12 (66.8)	16 (88.9)	0.504	6 (66.7)	21 (84.0)	0.140
Treatment failure	1 (5.5)	2 (11.1)		0 (0.0)	1 (4.0)	
Local recurrence	4 (22.2)	0 (0.0)		3 (33.3)	2 (8.0)	
Distant metastases	1 (5.5)	0 (0.0)		0 (0.0)	1 (4.0)	
Survival						
Alive at the last follow-up	12 (66.7)	16 (88.9)	0.102	3 (33.3)	19 (76.0)	0.072
Death from cancer disease	4 (22.2)	0 (0.0)		3 (33.3)	3 (12.0)	
Death from others reasons	2 (11.1)	2 (11.1)		3 (33.3)	3 (12.0)	

^a^Median value

^b^Row percentage

^c^Number of cigarettes per day × years of smoking

^d^Low level of drinking—no alcohol and occasional drinkers (at most two drinks a day, especially with a meal) high level of drinking—more than 15 drinks of high percentage alcohol in a week and alcoholics

Due to lack of material in 2 paraffin blocks, P16 expression was evaluated in the subgroup of 34 HPV16-positive patients (assessed by *E6* gene fragment amplification). There were 25 (73.5%) tumours with P16 overexpression and 9 (26.5%) without high protein expression (Table 2). P16 overexpression was significantly more frequent in cancers characterized by higher VL, and all cancers without P16 overexpression were characterized by lower VL. The distribution of tumours with P16 overexpression was also significantly associated with site of cancer localization, lymph node status and the level of drinking.

### HPV16 infection, viral load, P16 expression—survival analysis

For 109 patients, OS and DFS were 44.4 and 60.9%, respectively, and were significantly higher in patients with HPV16 infection (Table 3). Significantly better OS was also found for females and lower *T* and *N* stages. In turn, significantly higher DFS was noticed for females, patients with lower levels of smoking and drinking and with lower *T* stage and lower grade. All variables suggesting significantly better OS and DFS in univariate analysis were included in multivariate analysis. For OS, female gender, lower *N* stage and HPV16 presence were independent favourable prognostic factors (Table 4). For DFS, such factors proved to be: lower *T* stage, lower level of smoking and HPV16 presence.

**Table 3:** Univariate Cox proportional hazard model for 5-year overall and disease-free survival of 109 patients with squamous cell carcinoma of head and neck

	Overall survival				Disease-free survival			
	Response *N* (%)^a^	HR	95% CI	Log-rank *P*	Response *N* (%)^a^	HR	95% CI	Log-rank *P*
Age								
≤ 58 years^b^	22/51 (43.1)	1.354			33/51 (64.7)	1.119		
> 58 years	34/58 (58.6)	1.000	0.430–1.269	0.265	40/58 (69.0)	1.000	0.465–1.718	0.734
Gender								
Female	17/22 (77.3)	1.000			18/22 (81.8)	1.000		
Male	39/87 (44.8)	3.230	0.122–0.780	0.004	55/87 (63.2)	2.664	0.132–1.064	0.038
Status in the Karnofsky scale								
≤ 80%	21/49 (42.9)	1.712			29/49 (59.2)	1.912		
> 80%	35/60 (58.3)	1.000	0.340–1.004	0.051	44/60 (73.3)	1.000	0.271–1.011	0.052
Localization								
Oral cavity	10/24 (41.7)	5.962	0.781–45.497		15/24 (62.5)	2.034	0.439–9.418	
Oropharynx	34/68 (50.0)	4.458	0.614–32.829		48/68 (70.6)	1.329	0.310–5.693	
Hypopharynx	3/7 (42.9)	5.118	0.568–46.127		2/7 (28.6)	4.469	0.832–24.006	
Larynx	9/10 (90.0)	1.000		0.253	8/10 (80.0)	1.000		0.082
*T* stage								
1 + 2	18/26 (69.2)	1.000			22/26 (84.6)	1.000		
3 + 4	38/83 (45.8)	2.242	1.055–4.763	0.021	51/83 (61.4)	3.148	1.111–8.914	0.015
*N* stage								
0 + 1	23/37 (62.2)	1.000			27/37 (73.0)	1.000		
2 + 3	33/72 (45.8)	1.902	1.029–3.515	0.030	46/72 (63.9)	1.652	0.795–3.432	0.158
Grade								
1 + 2	53/96 (55.2)	1.000			68/96 (70.8)	1.000		
3	3/13 (23.1)	1.625	0.816–3.234	0.149	5/13 (38.5)	2.197	0.999–4.832	0.046
Keratinization								
Yes	27/62 (43.6)	1.734			38/62 (61.3)	1.863		
No	29/47 (61.7)	1.000	0.981–3.065	0.051	35/47 (74.5)	1.000	0.931–3.730	0.069
The level of smoking—Brinkman index^c^								
≤ 520^b^	32/54 (59.3)	1.000			42/54 (77.8)	1.000		
> 520	24/55 (43.6)	1.646	0.951–2.848	0.070	31/55 (56.4)	2.420	1.208–4.850	0.010
The level of drinking^d^								
Low	29/50 (58.0)	1.000			38/50 (76.0)	1.000		
High	27/59 (45.8)	1.588	0.914–2.760	0.094	35/59 (59.3)	2.106	1.051–4.221	0.030
HPV16 infection								
Present	23/36 (63.9)	1.000			30/36 (83.3)	1.000		
Absent	33/73 (45.2)	1.844	0.290–1.015	0.041	43/73 (58.9)	3.128	0.133–0.769	0.005
Treatment								
Definitive CRT or surgery + CRT	31/46 (67.4)	1.000			35/46 (76.1)	1.000		
Definitive RT or surgery + RT	16/38 (42.1)	1.778	0.919–3.439		25/38 (65.8)	1.531	0.686–3.418	
Induction CT + definitive RT	9/25 (36.0)	2.460	1.212–4.994	0.050	13/25 (52.0)	2.405	1.058–5.470	0.103

*HR* hazard ratio, *CI* confidence interval

^a^Row percentage

^b^Median values

^c^Number of cigarettes per day × years of smoking

^d^Low level of drinking—no alcohol and occasional drinkers (at most two drinks a day, especially with a meal) high level of drinking—more than 15 drinks of high percentage alcohol in a week and alcoholics

**Table 4:** Multivariate Cox proportional hazard model

	HR	95% CI	*P* value^a^
Overall survival—109 patients			
Gender			
Female	1.000		
Male	3.461	0.114–0.732	0.009
*N* stage			
0 + 1	1.000		
2 + 3	2.221	1.187–4.157	0.013
HPV16 infection			
Present	1.000		
Absent	2.134	0.247–0.887	0.020
Disease-free survival—109 patients			
*T* stage			
1 + 2	1.000		
3 + 4	3.229	1.137–9.170	0.028
The level of smoking—Brinkman index^b^			
≤ 520^c^	1.000		
> 520	2.149	1.069–4.319	0.032
HPV16 infection			
Present	1.000		
Absent	3.083	0.134–0.783	0.012
Disease-free survival—36 patients with HPV16 positivity (assessed by amplification of viral gene *E6* fragment)			
The level of smoking—Brinkman index^b^			
≤ 520^c^	1.000		
> 520	2.253	1.124–4.514	0.023
Viral load			
> 6764.3 copies/cell^c^	1.000		
≤ 6764.3 copies/cell	2.795	1.060–1.121	0.045

*HR* hazard ratio, *CI* confidence interval

^a^ *P* value was examined by the Cox proportional hazard model for multivariate survival analysis

^b^Number of cigarettes per day × years of smoking

^c^Median value

**Fig. 2:**
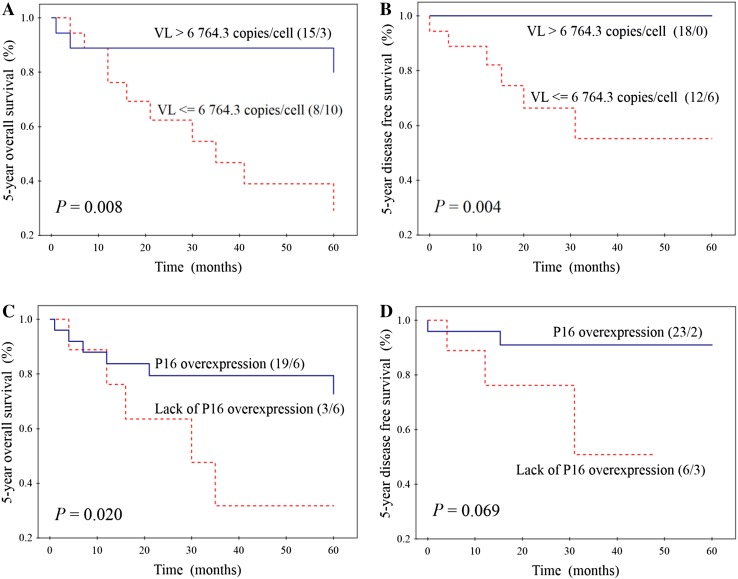
Correlations between HPV16 load (VL—the number of HPV16 copies per single cell) and overall survival (**a**) and disease-free survival (**b**) and between P16 expression and overall survival (**c**) and disease-free survival (**d**) in the subgroup of 36 patients with head and neck cancers with HPV16 positivity. Numbers in parentheses indicate the number of alive/dead patients or those with tumour regression/progression

Separate survival analysis was also performed in the subgroup of 36 patients with HPV16 positivity (assessed by *E6* gene fragment amplification). Better OS (Fig. 2a) and DFS (Fig. 2b) was significantly related to higher VL, wherein all patients with higher VL (*n* = 18) survived 5 years without any evidence of disease. Significantly higher OS was also found for patients having tumours with P16 overexpression than for those without overexpression (Fig. 2c). In case of DFS, this relation was similar, though not significant (Fig. 2d). OS was not significantly dependent on all epidemiological, clinical and histopathological features tested. In turn, higher DFS was also noticed for light smokers (95.0%) compared to heavy smokers (68.8%) (*P* = 0.034), as well as for patients with better performance status (95.2 vs 66.7%) (*P* = 0.021). Other features did not significantly influence DFS. Multivariate analysis was not performed for OS, because VL and P16 expression—two parameters, which significantly affected OS in univariate analysis—were significantly correlated with each other (Table 2). For DFS, in multivariate analysis three parameters were included: VL, the level of smoking and performance status. VL and the level of smoking were two independent prognostic factors for DFS of patients with HPV16 positivity (Table 4).

## Discussion

In the present study, among 109 patients with HNSCCs, HPV16 infection was found in 36 (33%) cases. It was also shown that using HPV16 load (the number of viral genome copies per single cell), it is possible to stratify patients with head and neck cancers and HPV16 positivity according to their prognosis. We have found, to the best of our knowledge for the first time, 100% DFS for patients with higher VL (median > 6764.3 copies/cell) (Fig. 2b). Moreover, multivariate analysis revealed this biomarker to be, beside the level of smoking, an independent prognostic factor for DFS of HPV16-positive patients. P16 expression represents less powerful prognostic factor than VL, because in this case statistical significance was achieved only for OS (Fig. 2c, d). Similar results were presented by Rödel et al. (2015), who in the group of 95 patients with anal HPV16-positive SCCs have shown significantly better local control and OS for patients with high VL and P16 overexpression. However, in their paper, VL and P16 expression did not significantly influence cancer-specific survival. Reports concerning HNSCCs have also shown significantly higher DFS for patients with tumours characterized by higher VL (Mellin et al. 2002; Cohen et al. 2008; Worden et al. 2008; Holzinger et al. 2012). We hypothesize that positive prognosis of patients with higher VL may be related to stimulation of immune response in the presence of transcriptionally active HPV infection. This hypothesis is supported by some studies in which correlations between higher VL and overproduction of circulating antibodies against plasma virus-like particles or antibodies against HPV16 oncoproteins E6 and E7 (Kreimer et al. 2005) and between higher VL and expression of HPV E6/E7 mRNA were found (Jung et al. 2010; Holzinger et al. 2012; Deng et al. 2013). On the other hand, in the present paper we have found, like other authors (Chang et al. 2014; Sannigrahi et al. 2016), significant positive relation between HPV16 VL and P16 expression (Table 2). In normal cells, P16 plays a role as a regulator of cell cycle, through, in brief, inhibition of phosphorylation of Rb family members (Serrano 1997). Because E7 oncoprotein (produced after integration of viral genome into the host genome) degrades Rb, P16 overexpression is observed in cells with transcriptionally active infection (Lewis et al. 2012). All these facts suggest higher activity of E6 and E7 in cells harbouring higher VL and stronger stimulation of systemic and local immune response. However, results of some studies showing positive correlation between *E6* expression and higher risk of recurrence contradict this hypothesis (Khwaja et al. 2016). It was also suggested that oropharyngeal SCCs with HPV infection were more likely to be B7-H1 positive (B7-H1 is involved in B7-H1/PD-1 signalling pathway of host immune suppression), which allowed to avoid inflammatory immune responses (Ukpo et al. 2013).

Despite above-mentioned results showing significant correlation between HPV16 VL and patients survival and hypothesis explaining these observations, prognostic potential of HPV16 VL in patients with HNSCCs is not clearly established. First of all, some authors (Huang et al. 2014) have shown opposite results, i.e. significantly higher rate of distant metastasis in patients with higher VL of HPV16 and HPV18. Besides, there are some controversies related to VL cut-off point which should be applied for optimal separation of survival curves. In the present paper, we have used median value (6764.3 copies/cell), in others it ranged from 15 (Huang et al. 2014) to 500 copies/cell (Cohen et al. 2008). These differences can be partly explained by analysis, in most of above-mentioned papers, of small patient group that was heterogeneous in respect to cancer localization. Besides, qPCR (used for VL estimation) may produce some divergent results, due to necessity of generation of reliable, reproducible standard curves (Roberts et al. 2008) or sample bias related to tumour “purity”, i.e. proportion of tumour cells to normal cells within sample, which can influence genetic analysis (Aran et al. 2015). Another explanation might be the use of fresh (Mellin et al. 2002; Worden et al. 2008) or archival, fixed material (Cohen et al. 2008; Huang et al. 2014). On the other hand, in our study, like in many other research works (Mellin et al. 2002; Cohen et al. 2008; Worden et al. 2008), VL is expressed as the number of viral genome copies per single cell, which allows to avoid discrepancies related to the quality of samples. Another controversy is related to the heterogeneity of analysed HNSCC patient group according to treatment type. In our study, patients with high VL were statistically significantly more often treated with cisplatin-based chemoradiotherapy (post-operative or independent) than patients having tumours with lower VL (Table 2). However, we did not decide to analyse the influence of treatment type on prognostic potential of VL due to the low number of cases in each subgroup. According to our best knowledge, there are also no data in the worldwide literature concerning this question in HNSCC patients. The study of Worden et al. (2008) included two subgroups of patients: one treated with induction chemotherapy followed by chemoradiotherapy and the second, in which, after induction chemotherapy and surgery, adjuvant radiotherapy was applied. In turn, Huang et al. (2014) analysed prognostic power of VL in the group of patients treated with radical surgery with or without adjuvant therapy (radiotherapy or chemoradiotherapy). In both these studies, prognostic power of VL was analysed irrespective of treatment type. Furthermore, the influence of treatment type was not the subject of analysis. Because high VL may reflect active HPV infection, we additionally reviewed available meta-analyses (Ragin and Taioli 2007; Dayyani et al. 2010; O’Rorke et al. 2012; Liu et al. 2016) in respect of testing the relation between prognostic significance of HPV presence and therapy regimens. In all meta-analyses, this relation was not tested, although all of them included studies involving different form of treatment (surgery alone or combination of surgery with induction or adjuvant radiotherapy or chemoradiotherapy). However, O’Rorke et al. (2012), discussing the results presented in their paper stated that the form of therapy may be one of the parameters confounding independence of HPV presence as risk factors. Taking all these facts into account, we think that VL has the potential to be reliable prognostic biomarker, although it requires validation in adequately large and homogeneous group of HNSCC patients according to tumour localization and treatment type.

Expression of P16 is a known surrogate marker of HPV infection (Serrano 1997). However, in the present paper we decided to analyse P16 immunostaining only in the subgroup of HPV16-positive tumours (identified on the basis of *E6* gene fragment amplification). The main reason for this decision is relative low specificity of P16 expression analysis which generates risk of false positive results (Prigge et al. 2017). In the subgroup of 36 HPV16-positive tumours, we identified 9 tissues with lack of protein overexpression and all these samples were characterized by lower VL (≤ 6764.3 copies/cell) (Table 2). On the contrary, among 25 cancers with P16 overexpression, there were 7 characterized by lower VL. These observations may be explained by the fact that overproduction of P16 can be caused not only by HPV infection, but also by oncogenes activation, DNA damage or accelerated cellular senescence (Li et al. 2011). In turn, genetic alteration of *P16* gene (deletion, methylation and point mutation), found in nearly 50% of malignancies, can inhibit synthesis of this protein (Li et al. 2011). Probably for these reasons, in the present paper P16 expression proved to be weaker prognostic marker than VL (Table 3). This finding is in line with those reported by other authors who have shown the best stratification of HNSCC patients with HPV positivity on the basis of VL and viral RNA expression (Jung et al. 2010; Holzinger et al. 2012).

Our results suggest that the better survival for HPV16-positive HNSCC patients is the effect of excellent prognosis of patients with higher viral load, which is probably related to transcriptionally active infection and stimulation of strong immune response. However, this assumption requires confirmation in further studies. Among HPV16-positive patients, viral load has stronger prognostic potential than P16 expression.
